# Supplementary material for: Association of Sarcopenia with Cognitive Function and Dementia Risk Score: A National Prospective Cohort Study
Source: Metabolites. 2023 Feb 8;13(2):245. doi: 10.3390/metabo13020245 (PMC9965467; doi:10.3390/metabo13020245)
Supplement: Supplementary file 1 [file metabolites-13-00245-s001.zip › metabolites-2125402-supplementary.pdf]

Supplemental materials

Table S1. Detailed algorithms of BDRM score

Items	Definition	Weight
1. Age	Age	1.09
2. History of stroke	We assigned a point of 1 to participants diagnosed with stroke; otherwise, we assigned a point of 0.	1.82
3. Subjective memory decline	Memory was estimated by the following questions: "How would you rate your memory at present?" (1) Excellent, (2) Very good, (3)Good, (4) Fair, and (5) Poor. Then we assigned a point of 1 to participants who answered "Poor"; otherwise, we assigned a point of 0.	1.31
4. Need for assistance with finances or medications	Needing for assistance with finances or medication was estimated by the following questions: "Do you have any difficulties with taking medications?" and "Do you have any difficulties with managing your money?" (1) No, I do not have any difficulty, (2) I have difficulty but can still do it, (3) Yes, I have difficulty and need help, and (4) I can not do it. Then we assigned a point of 1 to participants who chose (3) or (4); otherwise, we assigned a point of 0.	1.46

BDRM, Rotterdam Study Basic Dementia Risk Model.

Table S2. Association of sarcopenia and cognitive function after excluding participants with cognitive impairment or adjusting for cognitive function at baseline

	Model 1		Model 2	
	**β (95% CI)**	**P value**	**β (95% CI)**	**P value**
Sarcopenia (when excluding participants with cognitive impairment)				
No	Ref		Ref	
Yes	-0.39 (-0.52, -0.27)	<0.001	-0.16 (-0.26, -0.04)	0.005
Sarcopenia (when adjusting for cognitive function at baseline)				
No	Ref		Ref	
Yes	-0.12 (-0.22, -0.02)	0.013	-0.05 (-0.14, 0.03)	0.256

CI, confidence interval.

Model 1 was adjusted for age, gender, and follow-up time. Model 2 was additionally adjusted for marital status, education, residence, smoking status, alcohol consumption, CES-D score, BMI, and disease count.

Table S3. Association of sarcopenia and BDRM score after excluding participants with extremely high (i.e., >mean + 2SD) BDRM scores at baseline

	Model 1		Model 2	
	**β (95% CI)**	**P value**	**β (95% CI)**	**P value**
Sarcopenia				
No	Ref		Ref	
Yes	0.26 (0.18, 0.33)	<0.001	0.21 (0.13, 0.28)	<0.001

CI, confidence interval.

Model 1 was adjusted for gender, and follow-up time. Model 2 was additionally adjusted for marital status, education, residence, smoking status, alcohol consumption, CES-D score, BMI, and disease count.

Table S4. Association of sarcopenia and its components with cognitive function in total sample of CHARLS (N=7715)

	Model 1		Model 2	
	**β (95% CI)**	**P value**	**β (95% CI)**	**P value**
Sarcopenia				
No	Ref		Ref	
Yes	-0.43 (-0.52, -0.34)	<0.001	-0.21 (-0.29, -0.14)	<0.001
Sarcopenia components				
Low muscle strength				
No	Ref		Ref	
Yes	-0.35 (-0.42, -0.29)	<0.001	-0.23 (-0.28, -0.18)	<0.001
Low muscle mass [a]				
No	Ref		Ref	
Yes	-0.55 (-0.60, -0.50)	<0.001	-0.26 (-0.31, -0.21)	<0.001
Low physical performance				
No	Ref		Ref	
Yes	-0.09 (-0.14, -0.04)	<0.001	-0.054 (-0.01, -0.011)	0.012

CI, confidence interval.
Model 1 was adjusted for age, gender, and follow-up time· Model 2 was additionally adjusted for marital status, education, residence, smoking status, alcohol consumption, CES-D score, BMI, and disease count.
[a] Since age was used to define low muscle mass, we did not adjust for age in models.

Table S5. Association of sarcopenia and its components with BDRM score in total sample of CHARLS (N=7759)

	Model 1		Model 2	
	**β (95% CI)**	**P value**	**β (95% CI)**	**P value**
Sarcopenia				
No	Ref		Ref	
Yes	0.93 (0.83, 1.02)	<0.001	0.66 (0.57, 0.76)	<0.001
Sarcopenia components				
Low muscle strength				
No	Ref		Ref	
Yes	0.03 (0.02, 0.04)	<0.001	0.03 (0.01, 0.04)	<0.001
Low muscle mass [a]				
No	Ref		Ref	
Yes	0.90 (0.85, 0.96)	<0.001	0.83 (0.77, 0.89)	<0.001
Low physical performance				
No	Ref		Ref	
Yes	0.02 (0.01, 0.03)	<0.001	0.01 (0.005, 0.02)	0.003

CI, confidence interval.

Model 1 was adjusted for gender, and follow-up time· Model 2 was additionally adjusted for marital status, education, residence, smoking status, alcohol consumption, CES-D score, BMI, and disease count.

[a] Since age was used to define low muscle mass, we did not adjust for age in models.

Table S6. The association of sarcopenia and its components with cognitive function by gender

	Model 1		Model 2	
	**β (95% CI)**	**P value**	**β (95% CI)**	**P value**
**Male:**				
Sarcopenia				
No	Ref		Ref	
Yes	-0.47 (-0.63, -0.31)	<0.001	-0.22 (-0.38, -0.08)	<0.001
Sarcopenia components				
Low muscle strength				
No	Ref		Ref	
Yes	-0.41 (-0.53, -0.29)	<0.001	-0.27 (-0.38, -0.16)	<0.001
Low muscle mass [a]				
No	Ref		Ref	
Yes	-0.40 (-0.51, -0.29)	<0.001	-0.15 (-0.28, -0.02)	0.023
Low physical performance				
No	Ref		Ref	
Yes	-0.40 (-0.64, -0.18)	0.001	-0.22 (-0.42, -0.03)	0.024
**Female:**				
Sarcopenia				
No	Ref		Ref	
Yes	-0.29 (-0.48, -0.10)	<0.001	-0.06 (-0.21, 0.10)	0.400
Sarcopenia components				
Low muscle strength				
No	Ref		Ref	
Yes	-0.29 (-0.44, -0.15)	<0.001	-0.16 (-0.26, -0.04)	0.007
Low muscle mass				
No	Ref		Ref	
Yes	-0.41 (-0.53, -0.28)	<0.001	-0.20 (-0.33, -0.07)	0.003
Low physical performance				
No	Ref		Ref	
Yes	-0.32 (-0.67, 0.023)	0.068	-0.16 (-0.42, 0.10)	0.237

CI, confidence interval. Model 1 was adjusted for age and follow-up time. Model 2 was additionally adjusted for marital status, education, residence, smoking status, alcohol consumption, CES-D score, BMI, and disease count. [a] Since age was used to define low muscle mass, we did not adjust for age in models.

Table S7. The association of sarcopenia and its components with BDRM score by gender

	Model 1		Model 2	
	**β (95% CI)**	**P value**	**β (95% CI)**	**P value**
**Male:**				
Sarcopenia				
No	Ref		Ref	
Yes	0.51(0.33, 0.68)	<0.001	0.46 (0.28, 0.64)	<0.001
Sarcopenia components				
Low muscle strength				
No	Ref		Ref	
Yes	0.08 (0.03, 0.13)	<0.001	0.09 (0.04, 0.14)	0.001
Low muscle mass [a]				
No	Ref		Ref	
Yes	0.37 (0.26, 0.49)	<0.001	0.47 (0.31, 0.63)	<0.001
Low physical performance				
No	Ref		Ref	
Yes	-0.02 (-0.11, 0.07)	0.652	-0.01 (-0.01, 0.08)	0.841
**Female:**				
Sarcopenia				
No	Ref		Ref	
Yes	0.60 (0.41, 0.78)	<0.001	0.38 (0.19, 0.56)	<0.001
Low muscle strength				
No	Ref		Ref	
Yes	0.04 (-0.03, 0.11)	0.294	0.03 (-0.04, 0.10)	0.427
Low muscle mass				
No	Ref		Ref	
Yes	0.38 (0.26, 0.50)	<0.001	0.31 (0.15, 0.47)	<0.001
Low physical performance				
No	Ref		Ref	
Yes	-0.03 (-0.19, 0.13)	0.684	-0.02 (-0.18, 0.13)	0.767

CI, confidence interval. Model 1 was adjusted for age and follow-up time. Model 2 was additionally adjusted for marital status, education, residence, smoking status, alcohol consumption, CES-D score, BMI, and disease count. [a] Since age was used to define low muscle mass, we did not adjust for age in models.
